# Hypertensive response to exercise: mechanisms and clinical implication

**DOI:** 10.1186/s40885-016-0052-y

**Published:** 2016-07-26

**Authors:** Darae Kim, Jong-Won Ha

**Affiliations:** Cardiology Division, Severance Cardiovascular Hospital, Yonsei University College of Medicine, 50 Yonsei-ro, Seodaemun-gu, Seoul, 120-752 Republic of Korea

**Keywords:** Hypertension, Exercise

## Abstract

A hypertensive response to exercise (HRE) is frequently observed in individuals without hypertension or other cardiovascular disease. However, mechanisms and clinical implication of HRE is not fully elucidated. Endothelial dysfunction and increased stiffness of large artery contribute to development of HRE. From neurohormonal aspects, excess stimulation of sympathetic nervous system and augmented rise of angiotensin II seems to be important mechanism in HRE. Increasing evidences indicates that a HRE is associated with functional and structural abnormalities of left ventricle, especially when accompanied by increased central blood pressure. A HRE harbors prognostic significance in future development of hypertension and increased cardiovascular events, particularly if a HRE is documented in moderate intensity of exercise. As supported by previous studies, a HRE is not a benign phenomenon, however, currently, whether to treat a HRE is controversial with uncertain treatment strategy. Considering underlying mechanisms, angiotensin receptor blockers and beta blockers can be suggested in individuals with HRE, however, evidences for efficacy and outcomes of treatment of HRE in individuals without hypertension is scarce and therefore warrants further studies.

## Background

Systolic blood pressure (BP) normally rises with exercise as cardiac output increases during exercise in responses to the increased demand of oxygen from working muscles via increased sympathetic tone. However, some individuals present with abnormally exaggerated rise in systolic BP during exercise. This phenomenon is known as a hypertensive response to exercise (HRE). Yet, there is no consensus about exact value of systolic BP to define a HRE. Previous studies defined HRE as a difference between peak and baseline systolic BP at least 60 mmHg in men and at least 50 mmHg in women during exercise testing or systolic BP exceeding the 90^th^ percentile (approximately a systolic BP > 210 mmHg in men and >190 mmHg in women) [1–3].

A HRE is often observed in individuals without known cardiovascular diseases. Although it is generally considered as an abnormal response, there are conflicting data regarding its clinical significance. Some studies suggest that a HRE is associated with future development of hypertension and predict cardiovascular mortality [4–6], while others have not found such associations [7, 8]. In this review, we aimed to describe possible pathophysiologic mechanisms of HRE and its clinical implications.

### Mechanisms of hypertensive response to exercise

Although pathophysiology of HRE has not been fully understood, there are plausible mechanisms to explain this phenomenon. From a mechanistic point of view, a HRE can be explained by impairment of exercise induced endothelial vasodilation [9, 10]. Endothelium-dependent vasodilation in conduit arteries occurs in response to systolic wall sheer stress during exercise. Impaired endothelial function may limit vasodilation in response to increased shear stress from exercise, therefore, result in HRE. Indeed, Wilson et al. [11] showed significantly higher peripheral resistance at all stages of exercise observed in normotensive individuals with HRE. Consistent with previous studies, Stewart et al. [10] demonstrated an independent correlation between flow mediated dilation and exercise BP in normotensive population, suggesting that impaired endothelial vasodilation may contribute to exercise hypertension. Reduced nitric oxide (NO) activity was demonstrated in patients with HRE, even in young subjects without overt cardiovascular risk factors [12].

While the impairment of endothelial function mainly contributes to HRE in younger individuals, arterial stiffness should be considered as the mechanism of HRE in older population. Increased arterial stiffness in elderly results in a reduction in arterial compliance, followed by a reduction of buffering capacity of BP, and finally lead to an abnormal increase in BP during exercise. Although a few studies report conflicting data, previous studies demonstrated positive association of HRE with large artery stiffness, assessed by pulse wave velocity, central pulse pressure, and mean arterial pressure [13].

During exercise, sympathetic nervous system and renin-angiotensin-aldosterone system (RAAS) play an important role, mediating heart rate and BP increments in normal healthy population [11, 14, 15]. We have demonstrated that augmented rise of angiotensin II during exercise in individuals with HRE when compared to age and gender matched control group with normal BP reactivity during exercise [1]. Plasma epinephrine and norepinephrine also increased in both groups of individuals with HRE or normal response during exercise. However, there was no significant difference between two groups. Augmented rise in angiotensin II in HRE group was consistently reproduced in following studies, although extents of increased levels were not the same, possibly due to different magnitude of exercise and sensitivity of measurements [12]. Importance of angiotensin II in HRE is also evidenced by a significant reduction of peak systolic BP during exercise with angiotensin II receptor blocker [16].

Other possible mechanisms, such as an abnormal glucose metabolism, insulin resistance, or endothelium derived hyperpolarizing factors, were suggested although investigated in a limited number of patients [17, 18].

### Clinical implication

Deleterious effects of HRE on structure and function of left ventricle (LV) has been reported consistently. Theoretically, individuals with HRE would be exposed to abnormally high pressure loads to left ventricle (LV), which may result in global subendocardial ischemia due to mismatch between demand and supply from excessive rate-pressure stress. Indeed, a previous study demonstrated a greater likelihood of new or worsening abnormalities of wall motions from echocardiography in individuals with HRE, even in the absence of angiographically significant coronary artery stenosis [19].

Many of previous studies demonstrated increased LV mass index and stiffness in HRE, evidenced by higher prevalence of LV hypertrophy and diastolic dysfunction from echocardiography [20–22]. However, it should be noted that some studies reported conflicting data, insisting that a HRE is not accompanied with diastolic dysfunction or LV remodeling compared to those with normal response to exercise [1, 23, 24].

**Fig. 1:**
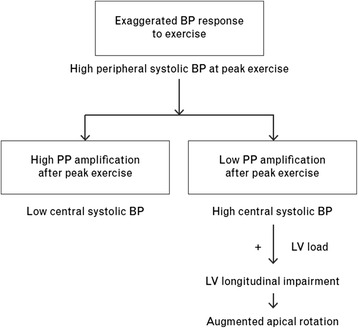
Pathophysiological pathways through which exercise pulse pressure amplification may contribute to the development of left ventricular longitudinal dysfunction in individuals with an exaggerated blood pressure response to exercise (adapted from ref [25])

While differences in modalities and intensities of exercise, or baseline characteristics of participants may be responsible for inconsistency in previous data, Shim et al. [25] provided an interesting possible explanation. In this study, central hemodynamic characters were assessed in individuals with HRE and compared to those with normal responses during exercise. Diverse central BP responses were observed in individuals: high pulse pressure amplification vs. low pulse pressure amplification after exercise. It implicates that a HRE is not always accompanied by elevation of central BP, which is a well- known factor to target organ damage and future cardiovascular events. Therefore, a HRE would result in abnormal LV function and structural remodeling in presence of arterial stiffening (Fig. 1).

A HRE is generally conceived as a risk for future development of hypertension, evidenced by a substantial number of previous studies with either bicycle or treadmill exercise [11, 26]. Systolic BP measured during light to moderate exercise is shown to predict masked hypertension with high specificity in individuals with HRE [27]. However, current guideline does not recommend exercise testing as a screen test for prehypertension given the low predictive value [28]. A previous meta-analysis revealed that a HRE at moderate exercise workload increased cardiovascular outcomes (fatal or non-fatal myocardial infarction, stroke, or development of coronary artery disease) by 36 % (95 % CI, 1.02-1.38, *p* = 0.039) after adjustment for other cardiovascular risk factors [29]. In this study, every 10 mmHg increase in systolic BP during exercise at moderate intensity was accompanied by a 4 % increase in cardiovascular event and mortality independent of mode of exercise. It is interesting that prognostic value of BP during high work load was not as influential as BP during moderate work load. Although future studies are warranted, submaximal workload may better reflect daily ambulatory BP of participants, therefore, clinically more relevant than BP during high work load. Also, difficulties in measuring BP during high work load may have attributed to such results.

### Treatment

As suggested by previous studies, exaggerated BP response to exercise is not a benign phenomenon, but it is still controversial whether to treat HRE in individuals without hypertension. Moreover, treatment strategy is still uncertain. Given previous data, angiotensin II is considered as the most important neurohormone affecting vascular and myocardial responses to exercise. Therefore, angiotensin receptor blocker or angiotensin converting enzyme inhibitor could be recommended as a treatment for individuals with HRE. Beta blocker could also be the drug of choice considering increased sympathetic tone during exercise. Currently, there is an ongoing clinical trial investigating efficacy of Fimasartan and Atenolol in hypertensive patients with HRE. Further results would provide insight to treatment strategy for HRE in individuals without hypertension.

## Conclusion

A HRE is pathologic phenomenon and associated with functional and structural impairment of LV, future development of hypertension and increased cardiovascular event, although conflicting data exist due to diverse reponses to central hemodynamics, various methodologies, and different clinical characteristics of study population. Further consensus on definition and method to measure HRE may provide insightful information to clinicians.

## Abbreviations

BP, blood pressure; HRE, hypertensive responses to exercise; LV, left ventricle; NO, nitric oxide; RAAS, renin-angiotensin-aldosterone system.
